# Intimate genetic relationships and fungicide resistance in multiple strains of *Aspergillus fumigatus* isolated from a plant bulb

**DOI:** 10.1111/1462-2920.15724

**Published:** 2021-08-31

**Authors:** Hiroki Takahashi, Sayoko Oiki, Yoko Kusuya, Syun‐ichi Urayama, Daisuke Hagiwara

**Affiliations:** ^1^ Medical Mycology Research Center Chiba University, 1‐8‐1 Inohana Chuo‐ku Chiba 260‐8673 Japan; ^2^ Molecular Chirality Research Center Chiba University, 1‐33 Yayoi‐cho Inage‐ku Chiba 263‐8522 Japan; ^3^ Plant Molecular Science Center Chiba University, 1‐8‐1 Inohana Chuo‐ku Chiba 260‐8675 Japan; ^4^ Faculty of Life and Environmental Sciences University of Tsukuba, 1‐1‐1 Tennodai Tsukuba Ibaraki 305‐8577 Japan; ^5^ Microbiology Research Center for Sustainability University of Tsukuba, 1‐1‐1 Tennodai Tsukuba Ibaraki 305‐8577 Japan

## Abstract

Fungal infections are increasingly dangerous because of environmentally dispersed resistance to antifungal drugs. Azoles are commonly used antifungal drugs, but they are also used as fungicides in agriculture, which may enable enrichment of azole‐resistant strains of the human pathogen *Aspergillus fumigatus* in the environment. Understanding of environmental dissemination and enrichment of genetic variation associated with azole resistance in *A*. *fumigatus* is required to suppress resistant strains. Here, we focused on eight strains of azole‐resistant *A*. *fumigatus* isolated from a single tulip bulb for sale in Japan. This set includes strains with TR_34_/L98H/T289A/I364V/G448S and TR_46_/Y121F/T289A/S363P/I364V/G448S mutations in the *cyp51A* gene, which showed higher tolerance to several azoles than strains harbouring TR_46_/Y121F/T289A mutation. The strains were typed by microsatellite typing, single nucleotide polymorphism profiles, and mitochondrial and nuclear genome analyses. The strains grouped differently using each typing method, suggesting historical genetic recombination among the strains. Our data also revealed that some strains isolated from the tulip bulb showed tolerance to other classes of fungicides, such as QoI and carbendazim, followed by related amino acid alterations in the target proteins. Considering spatial–temporal factors, plant bulbs are an excellent environmental niche for fungal strains to encounter partners, and to obtain and spread resistance‐associated mutations.

## Introduction

Azoles are versatile compounds that show outstanding activity against a wide range of fungi, including plant and human pathogens. These compounds play an essential role in agricultural and clinical settings as fungicides and antifungal drugs (Price *et al*., 2015; Fisher *et al*., 2018). Their main mode of action is inhibition of the ergosterol biosynthesis pathway by inhibiting Cyp51, which functions as a 14‐alpha‐demethylase critical for the biosynthesis of ergosterol. Azole fungicides, known as demethylase inhibitors (DMIs), include triazole and imidazole compounds such as tebuconazole, propiconazole, triflumizole and prochloraz. They are widely used to protect crops and fruits against pathogens by application during cultivation and postharvest preservation, as well as for seed disinfection. In medicine, azole drugs are essential options to combat dermatophytes and deep‐seated fungal pathogens, such as *Trichophyton rubrum* and *Aspergillus fumigatus* respectively. Azoles are the only class of compound used to control fungi in both agriculture and medicine.

*Aspergillus fumigatus* is a major causative agent of aspergillosis and is ubiquitously present in the environment as a saprobe. A limited number of antifungals are approved for therapy of *A*. *fumigatus* infection; voriconazole (VRCZ) and itraconazole (ITCZ) are the first‐line drugs for the treatment of pulmonary infection (Jenks and Hoenigl, 2018). However, this antifungal therapy is threatened by azole‐resistant *A*. *fumigatus*, strains of which have been increasingly isolated since the beginning of this century (Howard *et al*., 2009). The resistance mechanisms to azole drugs that have been identified in *A*. *fumigatus* from clinical settings are mutations in Cyp51A, HMG‐CoA reductase HMG1, and a subunit of CCAAT‐binding complex HapE, and overexpression of *cdr1B*, which encodes an ABC transporter (Camps *et al*., 2012; Fraczek *et al*., 2013; Hagiwara *et al*., 2016a; Hagiwara *et al*., 2018; Rybak *et al*., 2019; Hortschansky *et al*., 2020; Nywening *et al*., 2020). These azole resistance mutations are thought to have emerged during therapy with prolonged azole treatment.

However, in addition to treatment‐based resistance, environmentally derived resistance has been considered as a non‐negligible source of azole drug resistance of *A*. *fumigatus* during the last decade (Berger *et al*., 2017; Lestrade *et al*., 2019). Typical resistant strains from the environment carry a tandem repeat (TR) and single‐nucleotide polymorphisms (SNPs) in the promoter and coding regions of the *cyp51A* gene respectively. The most prevalent variants are TR_34_/L98H and TR_46_/Y121F/T289A, which were isolated for the first time from patients in Europe in 1998 and North America in 2008 respectively (Jeanvoine *et al*., 2020). The mutants with TR_34_ typically show high resistance to ITCZ, whereas the strains with TR_46_ show VRCZ resistance, but some are pan‐azole‐resistant strains. These genotypes were later recovered from different environments worldwide (Hagiwara *et al*., 2018; Resendiz *et al*., 2018; Schoustra *et al*., 2019). Diverse resistant mutants with TRs in the Cyp51A‐encoding gene have been reported (Table 1).

**Table 1:** Reported Cyp51A variants with tandem repeats in azole‐resistant *A*. *fumigatus*.

Cyp51A allele	Country	References
TR_34_/L98H	Many places	–
TR_46_/Y121F/T289A	Many places	–
TR_53_	Colombia	Alvarez‐Moreno *et al*. (2017)
TR_34_/L98H/S302N	The Netherlands	Schoustra *et al*. (2019)
TR_34_/L98H/F495I	The Netherlands	Schoustra *et al*. (2019)
TR_34_/L98H/L343H	The Netherlands	Schoustra *et al*. (2019)
TR_34_/L98H/E356V	The Netherlands	Schoustra *et al*. (2019)
TR_34_/L98H/S297T/F495I	The Netherlands	Schoustra *et al*. (2019) and Cao *et al*. (2020)
TR_34_/L98H/T289A/I364V/G448S	Japan	Nakano *et al*. (2020), this study
TR_46_/Y121F/T289A/I364V	The Netherlands	Schoustra *et al*. (2019)
TR_46_/Y121F/M172I/T289A/G448S	The Netherlands, Japan, Iran	Zhang *et al*. (2017), Nakano *et al*. (2020), Ahangarkani *et al*. (2020a), Ahangarkani *et al*. (2020b), Fraaije *et al*. (2020)
TR_46_/Y121F/T289A/S363P/I364V/G448S	The Netherlands, Japan	Nakano *et al*. (2020), Fraaije *et al*. (2020), Zhang *et al*. (2021), this study
TR^3^_46_/Y121F/M172I/T289A/G448S	The Netherlands, Japan	Zhang *et al*. (2017), Nakano *et al*. (2020)
TR^4^_46_/Y121F/M172I/T289A/G448S	The Netherlands	Zhang *et al*. (2021)
TR_92_/Y121F/M172I/T289A/G448S	The Netherlands	Zhang *et al*. (2021)

Recently, a possible environmental hot spot for azole‐resistant *A*. *fumigatus* was proposed (Zhang *et al*., 2017). The TR‐type mutants were prevalently isolated from agricultural compost containing azole fungicide residues, whereas azole‐free compost was dominated by azole susceptible *A*. *fumigatus* strains. This view was also supported in other studies (Schoustra *et al*., 2019; Zhang *et al*., 2021), indicating that azole‐resistant strains are enriched under the selective pressure of environmental azoles. The work by Zhang *et al*. also suggested that sexual reproduction plays an important role in developing and evolving new *cyp51A* alleles for drug resistance in compost (Zhang *et al*., 2017). Taking into consideration that TR‐type drug‐resistant *A*. *fumigatus* mutants show cross‐resistance to DMIs (Snelders *et al*., 2012), azole‐containing environmental niches may serve as evolutionary incubators through genetic recombination.

The propagation of azole‐resistant *A*. *fumigatus* has been studied in an epidemiological manner using microsatellite analysis by short tandem repeats for *A*. *fumigatus* (STR*Af*), which is a widely accepted intraspecies typing method with high‐resolution discriminatory power (de Valk *et al*., 2005). TR‐type mutant strains were spread worldwide. Some isolates from multiple countries were genetically closely related to each other and some had identical microsatellite patterns (Hagiwara *et al*., 2016b; Wang *et al*., 2018; Cao *et al*., 2020; Pontes *et al*., 2020). Besides such international propagation, intranational clonal expansion was also reported in several countries (Chowdhary *et al*., 2012; Ahangarkani *et al*., 2020a; Ahangarkani *et al*., 2020b). Recent population genomic studies revealed that the azole‐resistant strains are globally distributed. The isolates were divided into two broad clades, and TR mutants belong to the populations in an uneven manner (Sewell *et al*., 2019). These data suggest that azole resistance primarily expanded by asexual and sexual propagation from a limited number of ancestors with TR‐type mutation, rather than locally and independently emerging in each environment.

It was recently proposed that resistant *A*. *fumigatus* strains are transferred internationally via imported plant bulbs (Dunne *et al*., 2017). Plant bulbs produced in the Netherlands and sold in Ireland were contaminated with TR‐type *A*. *fumigatus* mutants. Similar cases were also reported by two independent Japanese groups (Hagiwara, 2020; Nakano *et al*., 2020); azole‐resistant *A*. *fumigatus* with diverse Cyp51A variants were isolated from plant bulbs that were imported from the Netherlands and sold in Japanese gardening shops. These studies suggest that the widespread of azole‐resistant *A*. *fumigatus* mutants is attributable in part to trade in agricultural products including plant bulbs.

In the present study, to further understand genetic variations in plant bulb–associated isolates, we focused on eight *A*. *fumigatus* strains that were co‐isolated from a single tulip bulb in a previous screening study (Hagiwara, 2020). Sensitivity to medical and agricultural azoles, as well as other classes of fungicides, was compared between the strains. Whole‐genome comparison of the eight strains showed several fragmental overlaps of their genomes, suggesting genetic recombination had occurred between strains in the single bulb. Our work indicates that plant bulbs are not only a vehicle for the pathogen but also a place where the pathogen can evolve its drug resistance.

## Results

### Variation of Cyp51A mutation in strains from a single bulb

In a previous study, eight strains of *A*. *fumigatus* were isolated from a single tulip bulb as different colonies (hereafter referred to as strains 3‐1‐A to 3‐1‐H) (Hagiwara, 2020). Strain 3‐1‐H has no TR or SNPs in *cyp51A*, whereas TR_34_ or TR_46_ occur in combination with various SNPs in the other seven strains (Table 2). Strains 3‐1‐A, 3‐1‐E, 3‐1‐F and 3‐1‐G have a typical variant, TR_46_/Y121F/T289A. Strain 3‐1‐D has mutations S363P, I364V and G448S as well as TR_46_/Y121F/T289A. Strains 3‐1‐B and 3‐1‐C have TR_34_/L98H and mutations T289A, I364V and G448S. Notably, TR_34_/L98H and G448S are known to play a role in azole resistance, and T289A is typically accompanied by TR_46_ (Hagiwara *et al*., 2016a). Thus, the Cyp51A of strains 3‐1‐B and 3‐1‐C showed complicated sequence variation, including three mutations related to azole resistance.

**Table 2:** Cyp51A variation and microsatellite typing of the strains in this study.

Strain ID	Cyp51A variation	2A	2B	2C	3A	3B	3C	4A	4B	4C
3‐1‐A	TR_46_/Y121F, T289A	10	20	8	44	9	10	8	10	7
3‐1‐B	TR_34_/L98H, T289A, I364V, G448S	23	10	9	35	9	6	8	10	18
3‐1‐C	TR_34_/L98H, T289A, I364V, G448S	23	10	9	35	9	6	8	10	18
3‐1‐D	TR_46_/Y121F, T289A, S363P, I364V, G448S	24	20	12	45	9	11	8	10	18
3‐1‐E	TR_46_/Y121F, T289A	26	20	12	36	9	22	8	14	31
3‐1‐F	TR_46_/Y121F, T289A	25	20	12	45	11	6	10	12	18
3‐1‐G	TR_46_/Y121F, T289A	23	10	9	36	9	6	12	10	7
3‐1‐H	wt	23	19	15	33	11	7	13	9	5

### Varied sensitivity to azoles in the strains from a single bulb

**Fig. 1:**
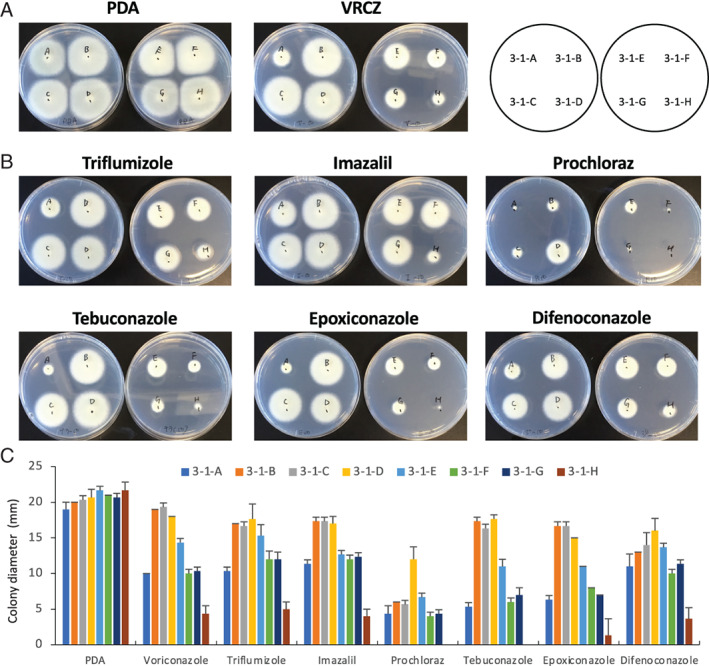
Colony growth of *A*. *fumigatus* strains isolated from a single tulip bulb on potato dextrose agar (PDA) containing azoles. A. Growth on PDA containing voriconazole (VRCZ). Each strain was inoculated on PDA with dimethylsulfoxide (DMSO) as a control or 10 μg ml^−1^ VRCZ and was incubated for 48 h. B. Growth on PDA containing demethylase inhibitors (DMIs). Each strain was inoculated on PDA with DMSO as a control or 10 μg ml^−1^ DMI and was incubated for 48 h. C. Colony diameter on PDA containing VRCZ or DMIs. Each strain was inoculated on PDA with DMSO as a control or 10 μg ml^−1^ azole and incubated for 28 h. Error bars represent standard deviations based on three independent replicates.

As previously reported, strains 3‐1‐A to G, which have TRs in *cyp51A*, showed VRCZ resistance (>32 μg ml^−1^) in minimum inhibitory concentration tests (Hagiwara, 2020). To further understand the susceptibility to azole drugs, colony growth was evaluated on potato dextrose agar (PDA) containing 10 μg ml^−1^ of VRCZ (Fig. 1A). Strains 3‐1‐B, 3‐1‐C and 3‐1‐D were more tolerant to VRCZ than the other strains. When grown on medium containing DMIs (triflumizole, imazalil, prochloraz, tebuconazole, epoxiconazole, or difenoconazole), strain 3‐1‐H, which harbours WT Cyp51A, showed the greatest growth inhibition among the strains. Strains 3‐1‐B, 3‐1‐C and 3‐1‐D were less affected by the DMIs (except prochloraz) (Fig. 1B). On the basis of colony diameter measurement, strains 3‐1‐B, 3‐1‐C and 3‐1‐D showed higher tolerance to VRCZ and DMIs than strains 3‐1‐A, 3‐1‐E, 3‐1‐F and 3‐1‐G (Fig. 1C). These results suggest that the combination of TR and G448S mutation increases resistance to azole compounds.

**Fig. 2:**
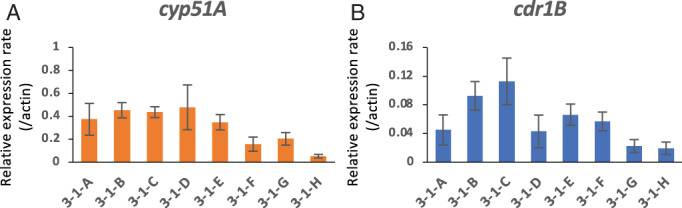
Gene expression analysis by quantitative real‐time (qRT)‐PCR. Expression levels of *cyp51A* (A) and *cdr1B* (B) were determined in the eight strains isolated from a single tulip bulb. The strains were cultured in potato dextrose broth for 18 h. The *actin* gene was used as an internal control. Error bars represent standard deviations based on three independent replicates.

The expression levels of genes related to azole resistance were examined in the eight strains by quantitative real‐time (qRT)‐PCR. Compared with strain 3‐1‐H, which has the WT *cyp51A* gene, strains with a TR in the *cyp51A* gene showed higher expression of *cyp51A* (Fig. 2A). Overexpression of *cdr1B*, which encodes an ABC transporter, has been reported to confer azole resistance. Thus, the expression level of *cdr1B* was also determined in the eight strains by qRT‐PCR. Strains 3‐1‐B and 3‐1‐C showed relatively high expression levels of *cdr1B* (Fig. 2B).

### Microsatellite typing analysis of tulip bulb isolates

**Fig. 3:**
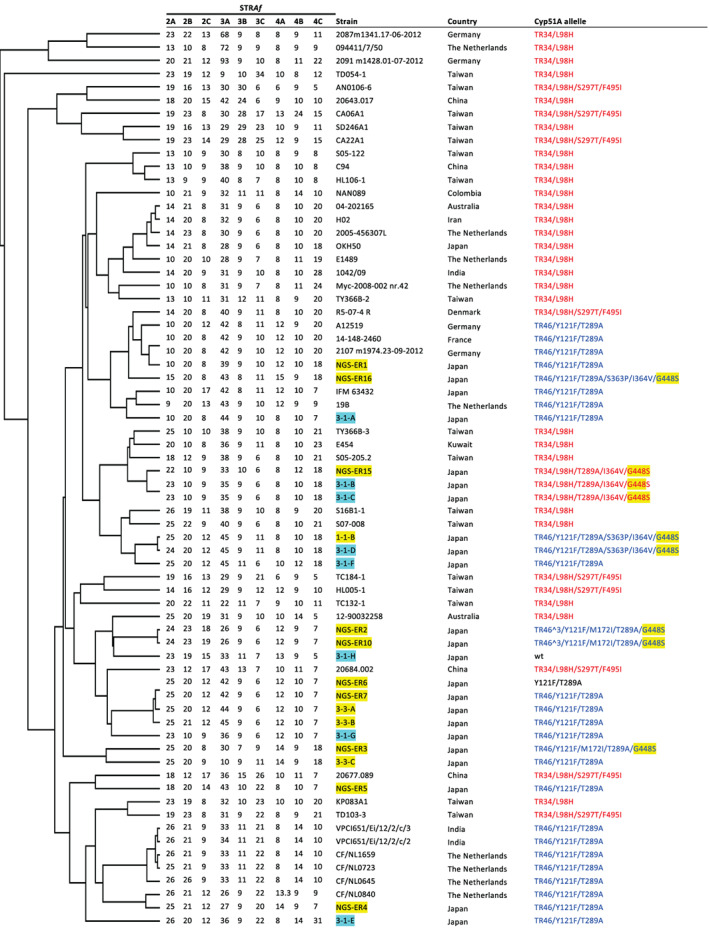
Microsatellite‐typing analysis of *A*. *fumigatus* strains with tandem repeat (TR) mutations. The dendrogram was constructed using short tandem repeat for *A*. *fumigatus* (STR*Af*) patterns of the strains. The nine STR panels are shown. The strains listed refer to the literature (Hagiwara *et al*., 2016b; Chen *et al*., 2019; Hagiwara, 2020; Nakano *et al*., 2020). The names of strains isolated from plant bulbs in this study or other study are highlighted in pale blue or yellow respectively. Cyp51A alleles with TR_34_ are indicated in red, and those with TR_46_ in blue.

As described in the Introduction, different Japanese group has isolated TR‐type azole‐resistant *A*. *fumigatus* strains from plant bulbs that were imported from the Netherlands (Nakano *et al*., 2020). To investigate the genetic relationships of plant bulb isolates between the different studies, microsatellite analysis using STR*Af* was performed (Table 2). Besides the 8 strains from a single tulip bulb, this analysis included 10 (NGS‐ER1, NGS‐ER16, NGS‐ER15, NGS‐ER2, NGS‐ER10, NGS‐ER6, NGS‐ER7, NGS‐ER3, NGS‐ER5 and NGS‐ER4) and four (1‐1‐B, 3‐3‐A, 3‐3‐B and 3‐3‐C) TR‐type strains that were previously reported in Nakano *et al*. (2020) and Hagiwara (2020) respectively (Fig. 3). In addition, TR‐type *A*. *fumigatus* isolated in different countries were also included from the literature (Hagiwara *et al*., 2016b; Chen *et al*., 2019). Among the eight strains from a single tulip bulb, the STR*Af* patterns of 3‐1‐B and 3‐1‐C matched perfectly. Strain 3‐1‐D is closely related to them, as this strain contains the same number of STRs in four of the nine panels. Similarly, strain 3‐1‐D shares the same number of STRs as strain 3‐1‐F in four of the nine panels. Interestingly, some strains that were isolated in Nakano *et al*. (2020) showed a close relationship with our strains. NGS‐ER15 had an STR pattern similar to that of our strains 3‐1‐B and 3‐1‐C (five of the nine panels), which is consistent with these strains having the same Cyp51A allele (TR_34_/L98H/T289A/I364V/G448S). Strains NGS‐ER6 and NGS‐ER7 are closely related to strains 3‐3‐A and 3‐3‐B that were isolated from a single another tulip bulb in our previous study (Hagiwara, 2020). Note that these extraordinarily close relatives were isolated from plant bulbs in different laboratories.

### Genome sequencing and comparison between strains

**Fig. 4:**
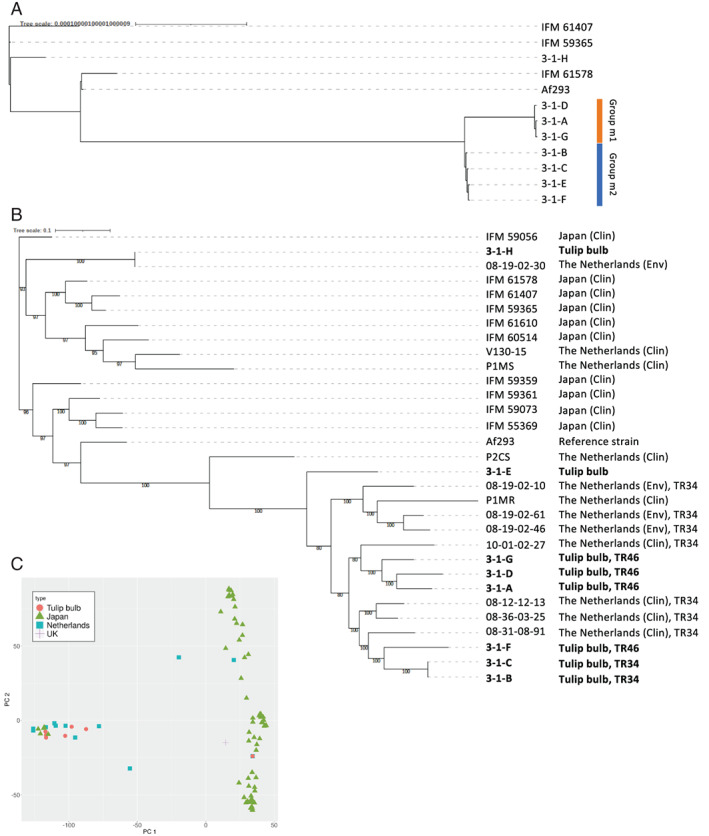
Phylogenetic trees constructed using mitochondrial (A) and nuclear (B) genomes. The trees were constructed using genomes of strains isolated from a single tulip bulb (3‐1‐A to 3‐1‐H) or clinically isolated in a previous study (Takahashi‐Nakaguchi *et al*., 2015) and shown in the literature (Fan *et al*., 2021), as well as *A*. *fumigatus* reference strain Af293. The clinical and environmental isolates are indicated as Clin and Env respectively. The types of tandem repeat in the *cyp51A* gene are depicted as TR34 or TR46 if the strain possesses the allele. C. Principal component analysis of 75 185 polymorphic loci of 96 strains was conducted. The strain and genome data used for the analyses are provided in Table S1.

To gain more insight into genetic differences or relatedness, genomes of the eight strains (3‐1‐A to H) were sequenced using the Illumina platform. Complete mitochondrial genomes were successfully obtained for the strains [31 749–31 770 base pairs (bp) long] (Table 3). A phylogenetic tree was constructed using the mitochondrial genomes and those of other strains (IFM 61407, IFM 59365 and IFM 61578) that had been clinically isolated in Japan (Takahashi‐Nakaguchi *et al*., 2015) (Fig. 4A). This dendrogram indicated that the eight strains isolated from the tulip bulb can be divided into three groups. Group m1 contains strains 3‐1‐A, 3‐1‐D and 3‐1‐G; strains 3‐1‐B, 3‐1‐C, 3‐1‐E and 3‐1‐F are in Group m2. Strain 3‐1‐H was distantly positioned from both Groups m1 and m2. Differences in the length of the mitochondrial genome well reflect the grouping, suggesting that strains within each group are very close relatives. In the microsatellite typing analysis described above, strains 3‐1‐B, 3‐1‐C, 3‐1‐D and 3‐1‐F were grouped into the same clade, but this was inconsistent with the grouping based on mitochondrial genomes, in which strain 3‐1‐D was not in the same group as strains 3‐1‐B, 3‐1‐C and 3‐1‐F.

**Table 3:** Results of genome sequencing for strains isolated from a single tulip bulb.

Strain ID	Total length of chromosomes (bp)	GC (%)	# of proteins	Mitochondrial genome (bp)	Mito group	CSP type	Mating type	SNP in *hmg1*
3‐1‐A	28 889 155	49.342	9492	31 770	m1	t02	*mat1‐1*	*E105K*, *S212P*, *Y564H*
3‐1‐B	28 519 682	49.352	9359	31 763	m2	t02	*mat1‐1*	*S212P*, *Y564H*
3‐1‐C	28 533 261	49.355	9490	31 763	m2	t02	*mat1‐1*	*S212P*, *Y564H*
3‐1‐D	29 087 830	49.324	9515	31 770	m1	t02	*mat1‐1*	*E105K*, *S212P*, *Y564H*
3‐1‐E	28 703 796	49.398	9464	31 763	m2	t02	*mat1‐1*	*S212P*, *Y564H*
3‐1‐F	28 808 510	49.492	9537	31 763	m2	t02	*mat1‐2*	*S212P*, *Y564H*
3‐1‐G	29 178 518	49.295	9543	31 770	m1	t02	*mat1‐1*	*E105K*, *S212P*, *Y564H*
3‐1‐H	28 716 638	49.611	9617	31 749	m3	t01	*mat1‐1*	*S212P*, *Y564H*

Nuclear genomes of the eight strains were compared with the reference genome of *A*. *fumigatus* strain Af293 (retrieved from AspGD, http://www.aspgd.org/); 92.2% to 93.6% of the Af293 genome was covered in the eight strains, and 69 943–79 384 SNPs were detected the genomes of the eight strains compared with the sequence of Af293 (Table 4). Phylogenetic analysis of the eight strains and previously sequenced strains including the isolates from Japan and the Netherlands was performed by using concatenated sequences of the SNP positions (Takahashi‐Nakaguchi *et al*., 2015; Fan *et al*., 2021) (Fig. 4B). Among the eight strains, 3‐1‐H was distantly positioned from the other seven strains (3‐1‐A to ‐G) in the dendrogram, whereas 3‐1‐H was very closely related to the Netherlands' environmental isolate 08‐19‐02‐30 (Abdolrasouli *et al*., 2015). The other seven strains showed moderately close genetic relatedness to each other and to the strains of the Netherlands, most of which have TR mutation. Strains 3‐1‐B and 3‐1‐C showed the closest relationship, which was supported by the largest number (75 484) of common SNPs against Af293 (Table 4). This is consistent with the results of microsatellite and mitochondrial genome typing. Nevertheless, in the mitochondrial genome typing, strain 3‐1‐E was in Group m2 with strains 3‐1‐B, 3‐1‐C and 3‐1‐F; however, strain 3‐1‐E was relatively distant from these three strains in phylogenetic analysis based on the nuclear genome (Fig. 4B).

**Table 4:** Summary of SNPs in strains isolated from a single tulip bulb.

	% of covered positions		# of common SNPs							
		# of SNPs	3‐1‐A	3‐1‐B	3‐1‐C	3‐1‐D	3‐1‐E	3‐1‐F	3‐1‐G	3‐1‐H
3‐1‐A	92.7%	74,858								
3‐1‐B	92.2%	77,709	56,717							
3‐1‐C	92.2%	77,873	56,852	75,484						
3‐1‐D	93.3%	78,387	60,722	58,958	59,009					
3‐1‐E	92.6%	76,211	53,708	52,539	52,712	50,947				
3‐1‐F	93.0%	75,546	57,638	57,673	57,825	54,169	51,842			
3‐1‐G	93.0%	79,384	62,638	63,534	63,599	64,147	52,463	57,704		
3‐1‐H	93.6%	69,943	29,767	33,662	33,632	34,092	32,742	31,065	33,711	

To gain deeper insight into geographic affiliation of the tulip isolates, principal component analysis of polymorphic loci was performed using total of 12 Netherlands and 75 Japanese isolates (Table S1). There were apparently different two populations one of which contains most of Japanese strains (Fig. 4C). In another population, seven of the tulip strains and Netherlands strains were included. These results suggested that the tulip bulbs had not been locally contaminated in Japan, but carried the strains from the Netherlands.

From the genome sequences, CSP typing was performed, which can typify strains by sequence variation at a single locus (*csp*: Afu3g08990) (Klaassen *et al*., 2009). The results showed that seven strains (3‐1‐A to 3‐1‐G) carried an identical type (t02), but strain 3‐1‐H had type t01. Sequence analysis for mating type revealed that all but strain 3‐1‐F harboured *mat1‐1*, whereas 3‐1‐F carried *mat1‐2* (Table 3).

The mutation in *hmg1* gene encoding a 3‐hydroxy‐3‐methylglutaryl‐coenzyme‐A reductase has been recently reported to be involved in resistance to triazoles (Rybak *et al*., 2019; Arai *et al*., 2021). From the genome sequences, SNPs in *hmg1* were extracted. There are E105K/S212P/Y564H in 3‐1‐A, ‐D and ‐G strains, whereas S212P/Y564H was found in the rest of strains (3‐1‐B, ‐C, ‐E, ‐F and ‐H) (Table 3). This classification was consistent with that of mitochondrial genome (Fig. 4A).

### Comparison of genome‐wide SNP frequency pattern

**Fig. 5:**
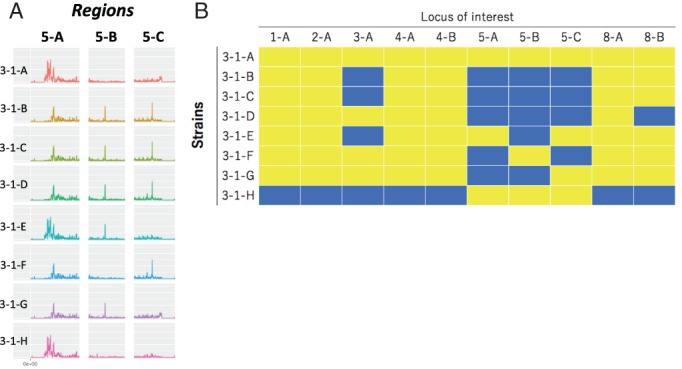
Differences in patterns of single nucleotide polymorphism (SNP) frequency among the strains isolated from a single tulip bulb. A. The SNP presence patterns are compared in certain regions on chromosome 5 (5‐A, 5‐B and 5‐C). B. The patterns in each region could typically be divided into two groups, which are indicated by yellow or blue panels. Ten genomic loci are shown and compared among the eight strains.

Inconsistency in strain typing among the typing methods using the mitochondrial and chromosomal genome sequences caused us to speculate that genetic recombination had occurred between the strains isolated from the single tulip bulb. To help test this hypothesis, the SNP frequency and distribution were investigated and compared among the strains in a genome‐wide manner (Fig. S1). There were several regions where the patterns of SNP frequency markedly differed among the strains (Fig. S1). For example, regions 5‐A, 5‐B and 5‐C on chromosome 5 were particularly characteristic (Fig. 5A). In region 5‐A, strains 3‐1‐A, 3‐1‐E and 3‐1‐H showed similar patterns of SNP frequency. In region 5‐B, the pattern of strain 3‐1‐A was similar to that of strains 3‐1‐F and 3‐1‐H. In region 5‐C, the pattern of strain 3‐1‐A was similar to that of strains 3‐1‐E, 3‐1‐G and 3‐1‐H. These results indicate that strain 3‐1‐A shares parts of the sequence of chromosome 5 with strains 3‐1‐E, 3‐1‐F, 3‐1‐G and 3‐1‐H. Such intergenomic variations were also found on other chromosomes (Figs 5B and S1). These results showed a genome‐wide mosaic pattern of SNP frequency, which is indicative of genetic recombination events in the strains.

### Comparing genome‐wide distribution of orthologous genes

To further investigate genome shuffling in the strains, we compared the patterns of orthologous among the strains isolated from the tulip bulb. First, the genes shared with the reference genome of *A*. *fumigatus* strain Af293 were investigated based on reciprocal blast hits (RBHs), which resulted in the isolates containing 8196 to 8322 orthologues of genes in strain Af293 (Table 5). The positions of the orthologues were generally evenly distributed in the strains, although fewer orthologues were found on chromosome 7. Notably, different patterns of orthologue content were displayed in some regions of the genomes of the various strains (Fig. 6). That is, some sets of strains have lost particular sets of genes, and other sets of genes have been lost in other sets of strains. Hence, the set of strains that shares an orthologue pattern is different at each locus (Fig. 6). This suggests repeated genome shuffling among the strains.

**Table 5:** The number of orthologous genes in the strains isolated from a single tulip bulb compared with reference strain *A*. *fumigatus* Af293.

		# of orthologous genes compared with strain Af293							
Chromosome	Af293	3‐1‐A	3‐1‐B	3‐1‐C	3‐1‐D	3‐1‐E	3‐1‐F	3‐1‐G	3‐1‐H
chr1	1642	1369	1355	1377	1371	1369	1365	1370	1370
chr2	1640	1433	1406	1425	1425	1411	1436	1427	1433
chr3	1395	1163	1163	1169	1179	1152	1173	1179	1189
chr4	1253	1089	1075	1093	1090	1098	1096	1085	1095
chr5	1367	1151	1156	1160	1153	1153	1162	1156	1153
chr6	1249	1068	1067	1074	1058	1069	1073	1070	1080
chr7	651	490	478	476	483	489	493	494	489
chr8	628	502	496	507	497	505	506	491	513
Total	9825	8265	8196	8281	8256	8246	8304	8272	8322

**Fig. 6:**
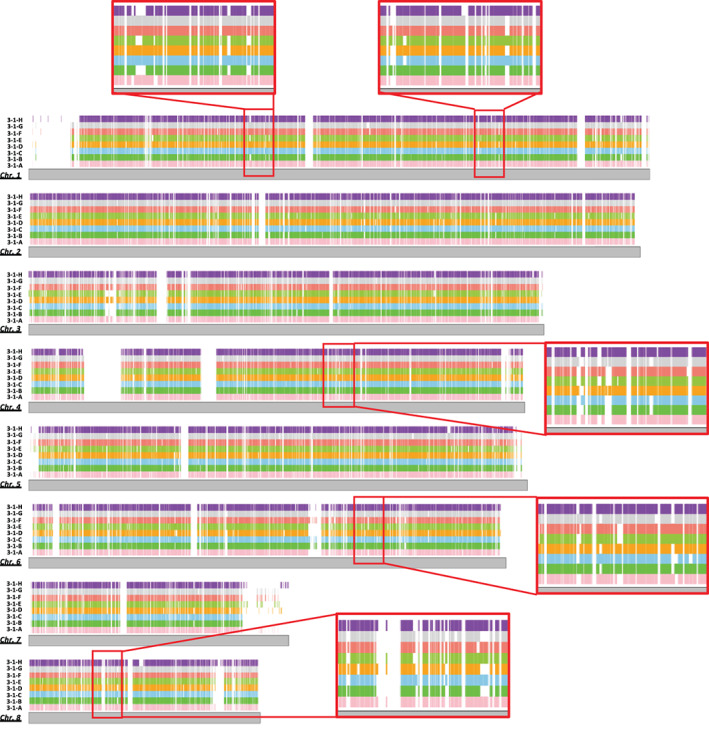
Visualization of genome‐wide orthologous gene content in the strains isolated from a single tulip bulb. Orthologous genes were searched against the reference genome of *A*. *fumigatus* strain Af293. The presence of an orthologue is indicated by coloured ribbons for each strain (3‐1‐A to 3‐1‐H). Some regions are enlarged to enable easier comparison of the patterns of gene content.

### Varied tolerance to agricultural fungicides

**Fig. 7:**
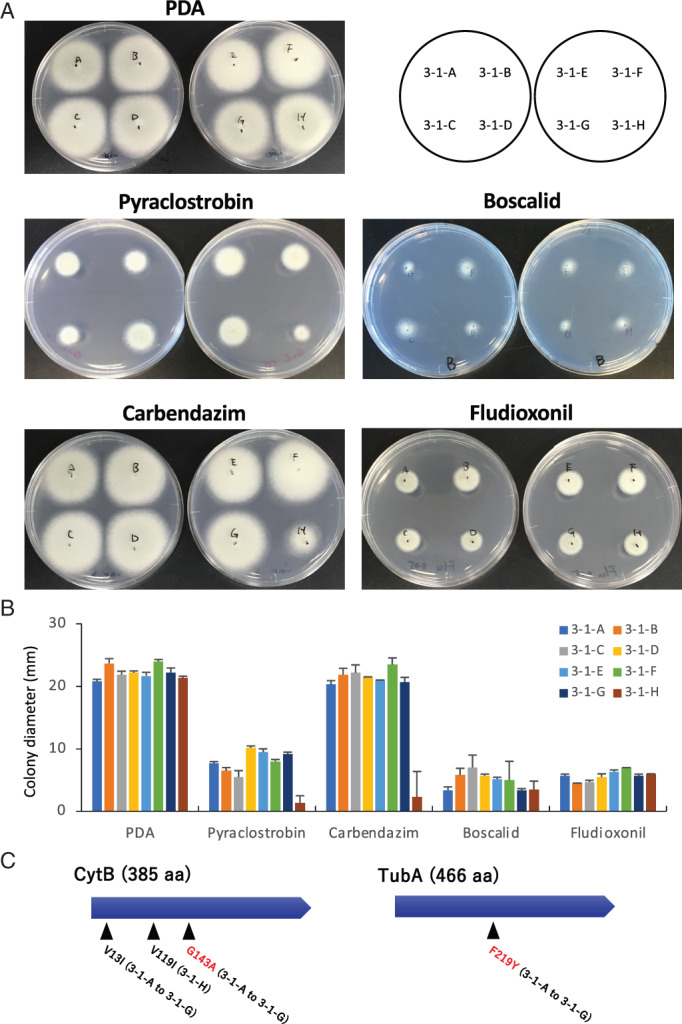
Colony growth of *A*. *fumigatus* isolated from a single tulip bulb on PDA containing fungicide. A. Growth on PDA containing fungicide. Each strain was inoculated onto PDA with DMSO as a control, or QoI (pyraclostrobin; 10 μg ml^−1^), SDHI (boscalid; 2.5 μg ml^−1^), methyl benzimidazole carbamate (carbendazim; 5 μg ml^−1^), or phenylpyrrole (fludioxonil; 0.2 μg ml^−1^), and incubated for 48 h. B. Colony diameter on PDA containing fungicide. Each strain was incubated for 30 h. Error bars represent standard deviations based on three independent replicates. C. Amino acid substitutions detected in CytB and TubA of *A*. *fumigatus* strains isolated from a tulip bulb.

As these strains were derived from a horticultural product, they may have been exposed to agricultural fungicides besides DMIs. Hence, the susceptibility of the eight strains to QoI (pyraclostrobin), SDHI (boscalid), methyl benzimidazole carbamate (carbendazim) and phenylpyrrole (fludioxonil) was evaluated on PDA plates. There was no significant difference among the strains in susceptibility to fludioxonil and boscalid (Fig. 7A and B). However, the colony of strain 3‐1‐H was smaller than those of the other seven strains on the medium containing pyraclostrobin or carbendazim. These results suggest that there is varied tolerance to pyraclostrobin and carbendazim among the strains.

From the genome sequences, mutations that are possibly responsible for tolerance to carbendazim and pyraclostrobin were searched in the target molecules tubulin and cytochrome *b* that are encoded by *tubA* (Afu1g10910) and *cytB* (AfuMt00001) respectively (Fig. 6C). The amino acid substitution F219Y was found in TubA of strains 3‐1‐A to 3‐1‐G. This substitution has been reported in several carbendazim‐resistant strains of plant pathogenic fungi (Yarden and Katan, 1993; Zhou *et al*., 2020). To investigate how the mutation is distributed in human pathogenic *A*. *fumigatus* genomes, the SNP database in FungiDB was explored. According to the dataset, 18% (14 of 77 strains) of *A*. *fumigatus* contain F219Y in TubA. Notably, eight of the 77 strains were isolated from the environment, and four of these possess the amino acid substitution.

In CytB, mutations V13I and G143A were found in strains 3‐1‐A to 3‐1‐G, and V119I was found in 3‐1‐H. G143A in CytB has been reported to confer the resistance to QoI in many plant pathogenic fungi (Samuel *et al*., 2011; Bolton *et al*., 2013), suggesting that this mutation in *A*. *fumigatus* is related to low sensitivity to QoI fungicide. As mitochondrial genome sequences are scarce in public databases, we investigated the sequence of the *cytb* gene in nine strains that were clinically isolated in a previous study and whose mitochondrial genome sequence is at least partly available (Takahashi‐Nakaguchi *et al*., 2015). Among these nine strains, no G143A mutation was observed, whereas seven of the strains contain V119I (as also observed in our strain 3‐1‐H).

## Discussion

The distribution of azole‐resistant *A*. *fumigatus* in natural environments has drawn increasing attention in recent years, with special interest in where the resistant strains have emerged, inhabit, and have been translocated to. However, deep understanding is still lacking. In this work, to fill in gaps in knowledge, we focused on strains that were isolated from a single tulip bulb.

Sexual reproduction of *A*. *fumigatus* was demonstrated in laboratory conditions in 2009 (O'Gorman *et al*., 2009). After this discovery, researchers paid more attention to the pan‐genome of clinical isolates of this pathogenic fungus. Population genetics study using linkage disequilibrium analysis for genetic markers supported the view that *A*. *fumigatus* reproduces asexually and sexually in natural habitats (Klaassen *et al*., 2012). This might accelerate gene flow among geographic regions, potentially blurring the geographic structure for some populations. Although there is accumulating evidence for genetic recombination in nature, proving the occurrence of sexual reproduction is difficult unless one can directly collect cleistothecia and ascospores formed in the environment. Genetic recombination in sexual development is suggested to cause the emergence of TR mutation in *cyp51A* gene through unequal crossover (Zhang *et al*., 2017). Thus, sexual reproduction is considered both to spread mutations by fusion with other strains and production of progeny and to locally produce *de novo* TR mutations, which could affect the prevalence of resistance to drugs and fungicides. Our data show that seven of the eight isolates from a single tulip bulb contain TR mutations, and the genetic variation between the TR‐containing strains is low compared with that between apparently independent strains. In addition, on the basis of genome‐wide distributions of SNPs and orthologous genes, genetic recombination is likely to have occurred between the seven strains.

Co‐isolation of the strains from a single bulb indicates that they have had much opportunities to physically interact with each other inside or on the bulb. In addition to the close spatial relationship of the fungal strains, they may interact for a long time. In the conventional process of plant bulb production, bulbs are multiplied from a parental bulb. This bulb multiplication is continued every year, which presumably causes the sustained presence of the fungi on/inside the bulbs. As described here, several strains were attached to a single bulb. These strains might have encountered others and genetically mixed many times. Once mutations giving rise to resistance to azoles emerged, the mutations could be preferentially and stably retained in the microbial community inside the bulb.

Sequencing analysis of the eight strains produced complete mitochondrial genomes and chromosomal genomes. The mitochondrial genome was of great help in interpreting whether there had been sexual reproduction among the strains. On the basis of mitochondrial genome sequences, the strains with TR mutation can be classed into Groups m1 and m2 (Fig. 4A). The length and sequences of the mitochondrial genomes are highly conserved in each group, indicating that they are genetically close progenies. However, the chromosomal genomes were diverse among the strains with TR mutation, excepting strains 3‐1‐B and 3‐1‐C which had approximately 97% of SNPs against Af293 in common (Table 4). The differences in grouping based on mitochondrial and chromosomal genomes strongly suggested a genetic recombination event. We therefore propose that the complete mitochondrial genome is valuable for gaining deeper insight into genetic relatedness among and between environmental and clinical isolates.

Strains with complicated *cyp51A* alleles have been reported in the literature and this article (Table 1). For instance, we have isolated *A*. *fumigatus* strains with TR_34_/L98H/T289A/I364V/G448S (3‐1‐B and 3‐1‐C) and TR_46_/Y121F/T289A/S363P/I364V/G448S (3‐1‐D) mutations in the *cyp51A* gene. TR_34_/L98H is a typical TR‐type mutation conferring resistance to ITCZ and in some cases to VRCZ, whereas TR_46_/Y121F/T289A confers resistance to VRCZ and in most cases to ITCZ (van Ingen *et al*., 2015; Buil *et al*., 2018). Amino acid substitution G448S contributes to resistance to VRCZ and occasionally to ITCZ (Bellete *et al*., 2010; Toyotome *et al*., 2016; Cao *et al*., 2020). Our finding that three strains (3‐1‐B, 3‐1‐C and 3‐1‐D) showed a higher tolerance to VRCZ and some DMIs than strains with only TR_46_/Y121F/T289A mutation is suggestive of elevation of tolerance to azole drugs by combining mutations. Importantly, strains with G448S mutation have been isolated not only from clinical samples but also from soil (Cao *et al*., 2020). We cannot rule out the possibility that the G448S mutation originally emerged and was retained in strains with TR_46_/Y121F/T289A under the selective pressure of fungicides.

The *A*. *fumigatus* strains used in the present work were isolated from a tulip bulb by culturing at 45°C on plates containing medium supplemented with fluconazole to select fungi that were resistant to fluconazole (Hagiwara, 2020). In total in that study, *A*. *fumigatus* was isolated from 50.8% of tulip bulbs (96/189), and strains isolated from 20.6% of the bulbs (39/189) had TR mutation. Because *A*. *fumigatus* is a saprophytic fungus that widely inhabits soil, compost, plant debris, wood chips, the air and aquatic environments, it was not surprising that half of the tulip bulbs were contaminated with *A*. *fumigatus*. However, we have no idea how the fungus resides on or inside the bulbs from a biological viewpoint. Because of the high frequency of *A*. *fumigatus* isolation from tulip bulbs, there might be certain mechanisms by which *A*. *fumigatus* colonizes and infects the plant tissue, enabling persistence across bulb progenies. Notably, some *A*. *fumigatus* strains were isolated from *Citrus macrocarpa*, *Myricaria laxiflora*, *Ligusticum wallichii* and *Moringa oleifera* (Arora and Kaur, 2019; Qin *et al*., 2019; Francisco *et al*., 2020; Li *et al*., 2020) as an endophyte. In general, however, the view that *A*. *fumigatus* has an endophytic mode in its life cycle remains to be established. In consideration of the dynamic mobilization of *A*. *fumigatus* in the environment, its association with plants may be overlooked, and we should pay more attention to it.

Recently, several field studies were published in which the prevalence of azole‐resistant *A*. *fumigatus* was investigated in association with fungicide use. Work by Zhou *et al*. (2021) demonstrated that the concentration of triazoles in the soil of greenhouses was not significantly correlated with azole susceptibility of isolates. In another study from Germany, a low frequency of azole‐resistant isolates from crop fields was reported regardless of azole fungicide use (Barber *et al*., 2020). A study by Fraaije *et al*. (2020) also reported a low number of azole‐resistant isolates in the soils of wheat‐cropping fields subjected to fungicide treatment. The authors considered that arable crop production is low risk for development of azole resistance. Conversely, a large‐scale survey across China was conducted, which showed that the residual level of azole fungicides in paddy soils positively correlated with the prevalence of azole‐resistant *A*. *fumigatus* (Cao *et al*., 2021). Field research on azole‐resistant *A*. *fumigatus* has started in many countries. More studies are required on the effects of fungicide use on the occurrence and spread of azole‐resistant *A*. *fumigatus* in the environment, including agricultural and horticultural settings.

In plant bulbs, there may be other pathogenic and nonpathogenic fungi beside *A*. *fumigatus*. They are also exposed to fungicides when the bulbs are treated with fungicide. Repeated use of fungicides would facilitate the occurrence of resistance mutations in non‐targeted fungi as well as in the target fungi of the pesticide. In the present study, we found that mutations in CytB and TubA that are related to resistance to QoI and carbendazim fungicides respectively, were detected in *A*. *fumigatus* strains as an example of non‐target fungi. These mutations might have been resulted from fungicide exposure during bulb production. Importantly, identical mutations of *A*. *fumigatus* were reported by Fraaije *et al*. (2020) and are found in database. These findings suggest that mutations related to resistance to antifungal agents are already present in the genomes of environmental fungi regardless of their pathogenicity. The boundary between acquired and natural resistance to antifungal compounds may become unclear in the near future.

## Materials and methods

### Strains and culture conditions

Strains 3‐1‐A to 3‐1‐H used in this study were obtained in previous study and were isolated from a single tulip bulb (Hagiwara, 2020). For plate and liquid cultures, PDA and PDB were used respectively. For colony growth tests, 10^5^ conidia of each strain were inoculated and incubated for 48 h at 37°C before taking pictures. In susceptibility tests, 10 μg ml^−1^ VRCZ, imazalil, prochloraz, triflumizole, tebuconazole, epoxiconazole and difenoconazole were respectively added to PDA. The control plate contained the equivalent volume of dimethylsulfoxide (DMSO). For measuring colony diameter, the culture time was 28 or 30 h. The data were obtained in triplicate, and the mean and standard deviation are presented. The fungicides fludioxonil, carbendazim, boscalid and pyraclostrobin were used at 0.2, 5, 2.5 and 10 μg ml^−1^ respectively.

### Quantitative real‐time RT‐PCR

Strains were cultured in PDB at 37°C for 18 h and harvested. The mycelia were frozen in liquid nitrogen, and total RNA was isolated using Sepasol Super G (Nacalai Tesque, Kyoto, Japan). cDNA was obtained by reverse transcription reaction using the total RNA sample and ReverTra Ace qPCR RT Master Mix with gDNA Remover (TOYOBO, Osaka, Japan).

Real‐time RT‐PCR was performed using Brilliant III Ultra‐Fast SYBR Green QPCR Master Mix (Agilent Technologies, Santa Clara, CA, USA) as described previously (Ninomiya *et al*., 2020). Relative expression ratios were calculated using the comparative cycle threshold (Ct) method. The actin‐encoding gene was used as a normalization reference. Each sample was tested in triplicate, and the standard deviation is presented. The primer sets used were described in Hagiwara *et al*. (2017).

### Microsatellite typing

Microsatellite typing was performed as described previously (Hagiwara *et al*., 2014). Briefly, nine microsatellite regions of approximately 400 bp were PCR amplified using purified genome DNA as a template and sequenced by the Sanger method. The repeat numbers of each locus were counted from the sequences. A dendrogram was constructed using Cluster 3.0 by hierarchical clustering with City‐block distance for average linkage and drawn using Treeview ver. 1.1.6r2 (de Hoon *et al*., 2004; Saldanha, 2004).

### Genome sequencing

Whole‐genome sequencing using next‐generation methods was performed as described previously (Hagiwara *et al*., 2018). In brief, we extracted genomic DNA from overnight‐cultured mycelia with NucleoSpin Plant II (Takara Bio, Shiga, Japan). For paired‐end library preparation, an NEBNext Ultra DNA Library Prep Kit (New England BioLabs, MA, USA) and NEBNext Multiplex Oligos (New England BioLabs) were used in accordance with the manufacturer's instructions. A total of 11 strains including 3‐1‐A to 3‐1‐H, IFM 59365, IFM 61407 and IFM 61578 were sequenced. Paired‐end sequencing (150‐bp) on a HiSeq 4000 system (Illumina, San Diego, CA, USA) was carried out by GENEWIZ (South Plainfield, NJ, USA).

### SNP detection

In addition to the abovementioned 11 strains, we used raw data for seven strains for comparison, which have been taken in a study by Takahashi‐Nakaguchi *et al*. (2015). Adapters and low‐quality bases from Illumina reads were trimmed by fastp (ver. 0.20.1) (Chen *et al*., 2018). Filtered reads were aligned against the *A*. *fumigatus* strain Af293 reference genome using BWA (ver. 0.7.17‐r1188) (Li and Durbin, 2009). SNP detection was performed as described previously (Hagiwara *et al*., 2018). Briefly, SNPs were identified by using SAMtools (ver. 1.9) (Li *et al*., 2009) and filtered with >20‐fold coverage, >30 mapping quality and 75% consensus using in‐house scripts (Tenaillon *et al*., 2012; Suzuki *et al*., 2014).

### Phylogenetic tree construction

Among the strains that were sequenced, mitochondrial genomes of 12 strains were available and aligned by MAFFT (ver. 7.475) (Katoh and Standley, 2013). A phylogenetic tree was constructed using multithreaded RAxML (ver. 8.2.12) (Stamatakis, 2014), the GTRCAT model, and 1000 bootstrap replicates, and visualized by iTOL (Letunic and Bork, 2019). For the genome‐wide phylogenetic analysis of 31 and 96 strains, Genome Analysis ToolKit (GATK) (ver. 4.1.2.0) (McKenna *et al*., 2010) was applied to detect the polymorphic loci on the chromosomal genomes according to Zhao *et al*. (2021). Then, 82 014 polymorphic loci of 31 strains were used for construction of a phylogenetic tree by the methods described above. Principal component analysis of 75 185 polymorphic loci of 96 strains was conducted by TASSEL (ver. 5.2.73) (Bradbury *et al*., 2007). A list of the strains and genomes used for the analyses is provided in Table S1.

### Genome assembly and gene prediction

Mitochondrial genomes were assembled and annotated using GetOrganelle (ver. 1.6.4) (Jin *et al*., 2020) and MITOS2 (Bernt *et al*., 2013) respectively. To filter the mitochondrial reads, trimmed reads were aligned against mitochondrial genomes by BWA (ver. 0.7.17‐r1188) (Li and Durbin, 2009), and the mapped reads were filtered by SAMtools (ver. 1.9) (Li *et al*., 2009) and SeqKit (Shen *et al*., 2016). Contigs were assembled by VelvetOptimiser (ver. 2.2.6) (Zerbino and Birney, 2008), followed by generation of a simulated mate‐paired library using wgsim (ver. 0.3.1‐r13) (https://github.com/lh3/wgsim). The assembly of nuclear genomes was carried out by ALLPATHS‐LG (ver. R52488) (Gnerre *et al*., 2011). The annotation of assembled nuclear genomes was performed by the Funannotate pipeline (ver. 1.7.4) (https://funannotate.readthedocs.io/en/latest/) as described previously (Takahashi *et al*., 2021). Following identification of repeat sequences by RepeatModeler (ver. 1.0.11) (http://www.repeatmasker.org/RepeatModeler.html) and RepeatMasker (ver. 4.0.7) (https://www.repeatmasker.org), Funannotate *ab initio* prediction was performed with the option ‘‐‐busco_seed_species = aspergillus_fumigatus’ by Augustus (ver. 3.3.3) (Stanke *et al*., 2006), GeneMark‐ES (ver. 4.38) (Ter‐Hovhannisyan *et al*., 2008), GlimmerHMM (ver. 3.0.4) (Majoros *et al*., 2004) and SNAP (ver. 2006‐07‐28) (Ian, 2004) using exon hints from the proteins of *A*. *fumigatus* Af293 and *N*. *fischeri* NRRL 181 downloaded from the *Aspergillus* Genome Database (http://www.aspgd.org/) (Cerqueira *et al*., 2014). The completeness of draft genomes and predicted proteins was evaluated by BUSCO (ver. 4.0.6) (Seppey *et al*., 2019) with the database eurotiales_odb10. Most tools were obtained through Bioconda (Grüning *et al*., 2018).

### Detection of orthologous genes

Orthologous relationships with *A*. *fumigatus* Af293 were determined by RBH with criteria BLASTp (ver. 2.9.0+) coverage >80% and identity >80% (Camacho *et al*., 2009).

### Visualization of genome‐wide distribution of SNPs and orthologous genes

SNP frequency in each 1‐kb window was calculated and plotted in 250‐bp steps using Python (Van Rossum and Drake, 2009) and R (R Core Team, 2019) scripts. The orthologous genes of *A*. *fumigatus* Af293 in each strain were visualized by R script.

## Author Contributions

H.T. and D.H. designed the research; H.T., S.O., Y.K., S.‐i.U. and D.H. performed experiments; H.T. contributed new materials/tools; H.T. and D.H. analysed data; and H.T. and D.H. wrote the manuscript.

## Supporting information


**Fig. S1.** Genome‐wide SNP frequency compared among the eight strains (3‐1‐A to 3‐1‐H). The 10 regions where the pattern is characteristically distinct among the strains are marked by red boxes.

**Table S1.** List of strains used for SNP typing analyses

## Data Availability

The genome sequencing data are deposited to DDBJ as DRA011961. BioSample accession(s): SAMD00322244–SAMD00322251.
