# Supplementary figures and images for: Intimate genetic relationships and fungicide resistance in multiple strains of Aspergillus fumigatus isolated from a plant bulb
Source: Environ Microbiol. 2021 Aug 31;23(9):5621–38. doi: 10.1111/1462-2920.15724 (PMC9292267; doi:10.1111/1462-2920.15724)

A

Chr.1

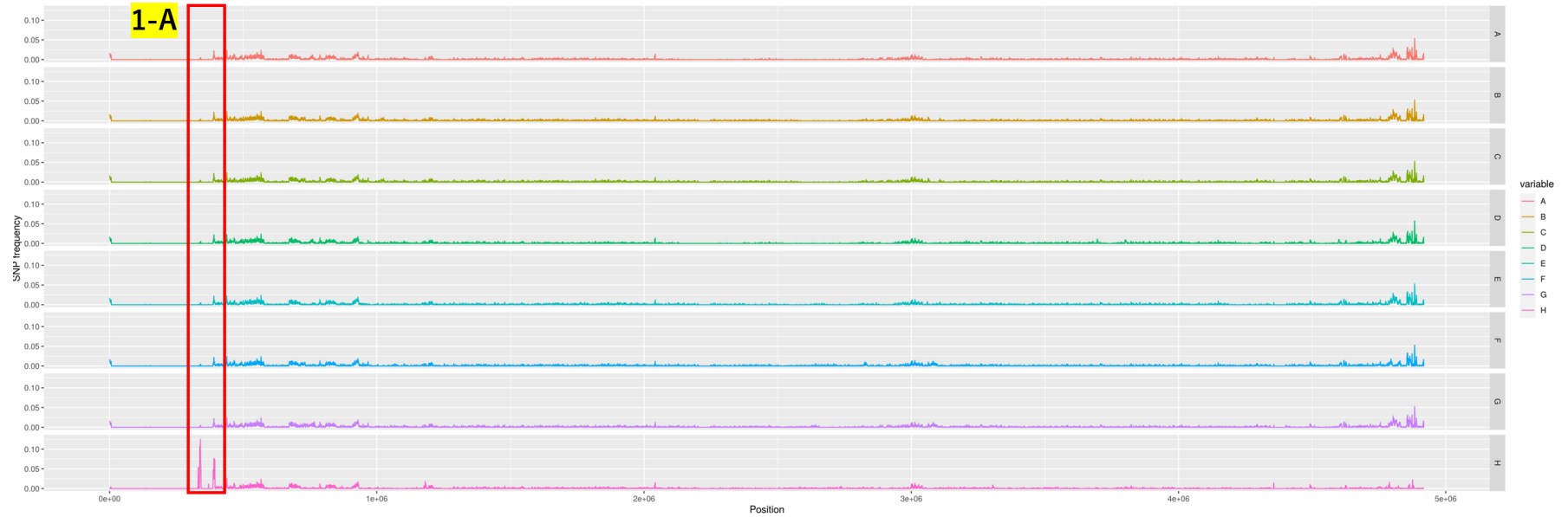

B

Chr.2

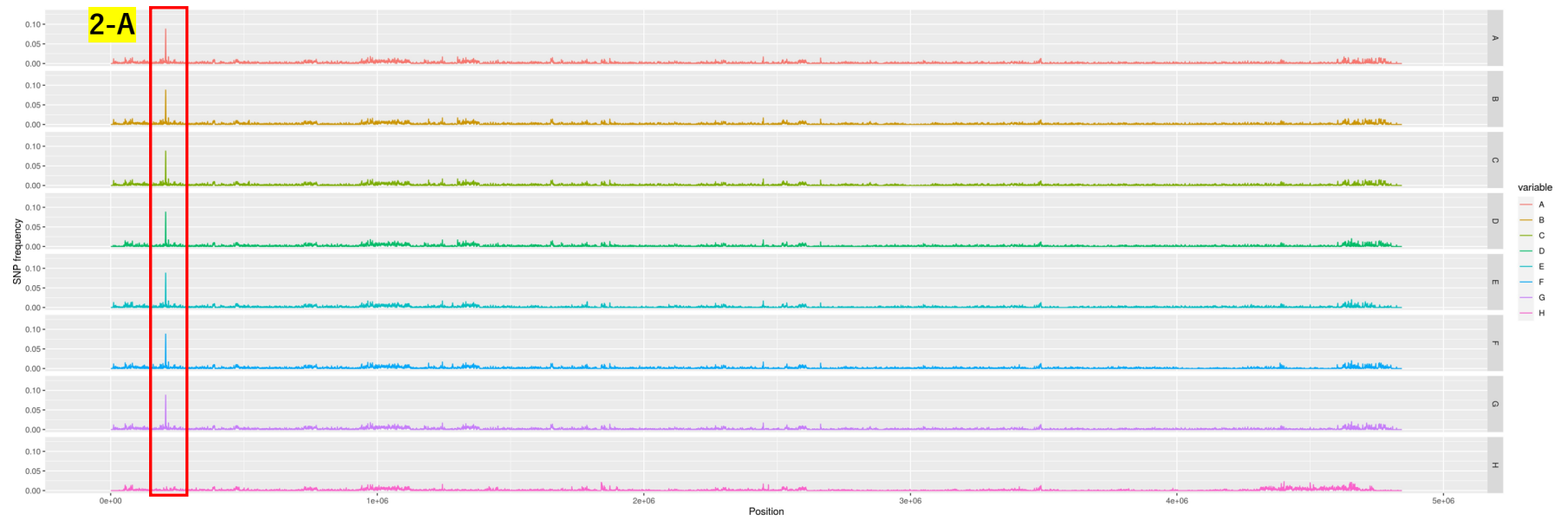

C

Chr.3

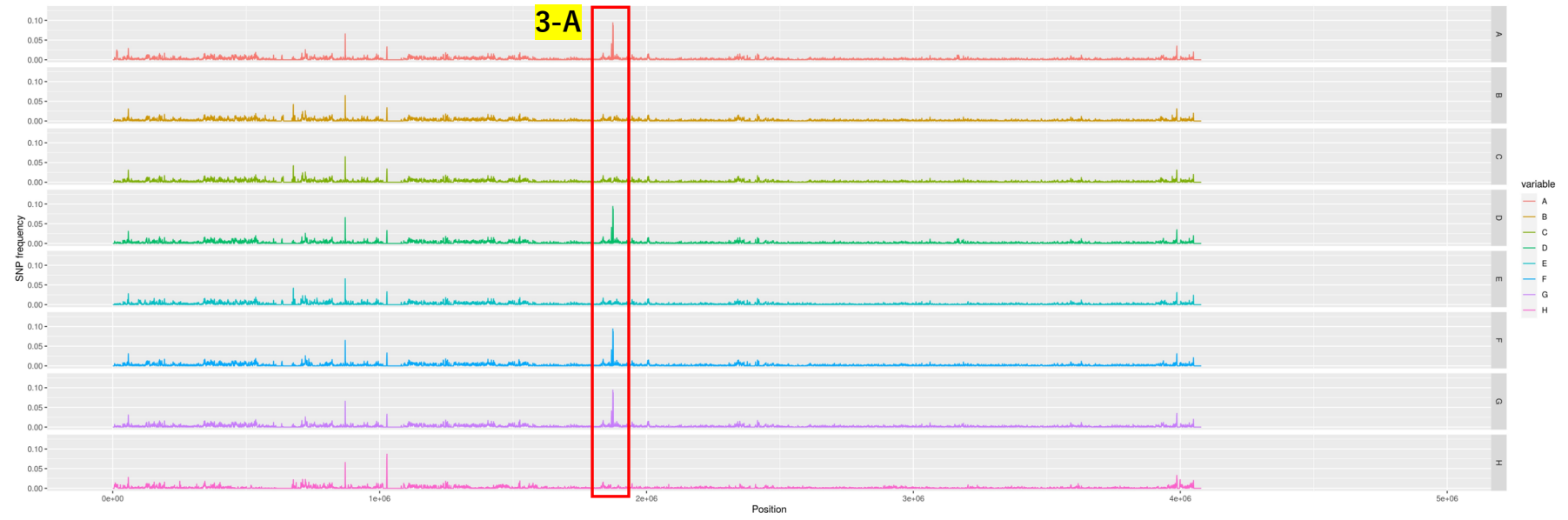

D

Chr.4

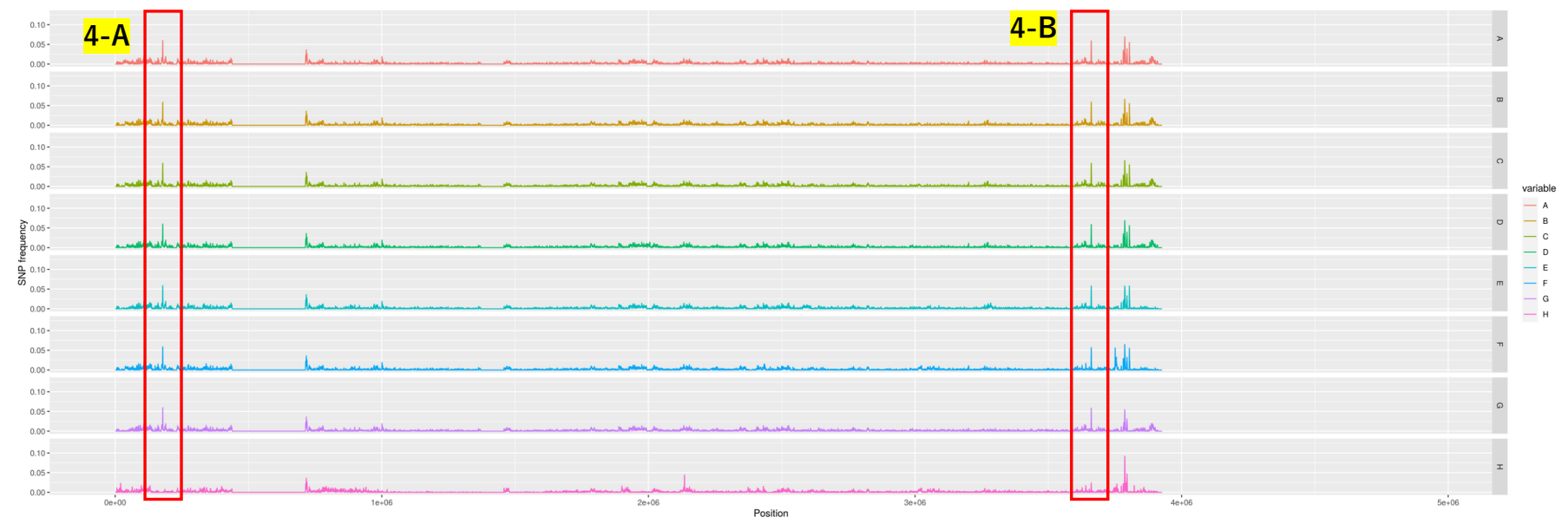

E

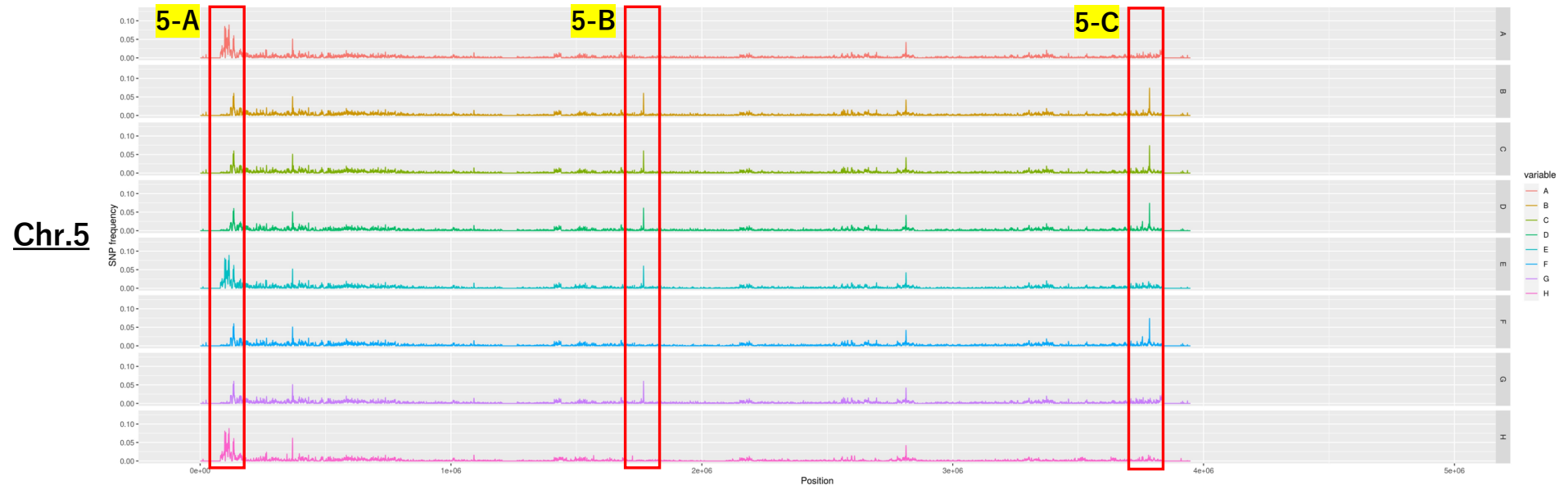

F

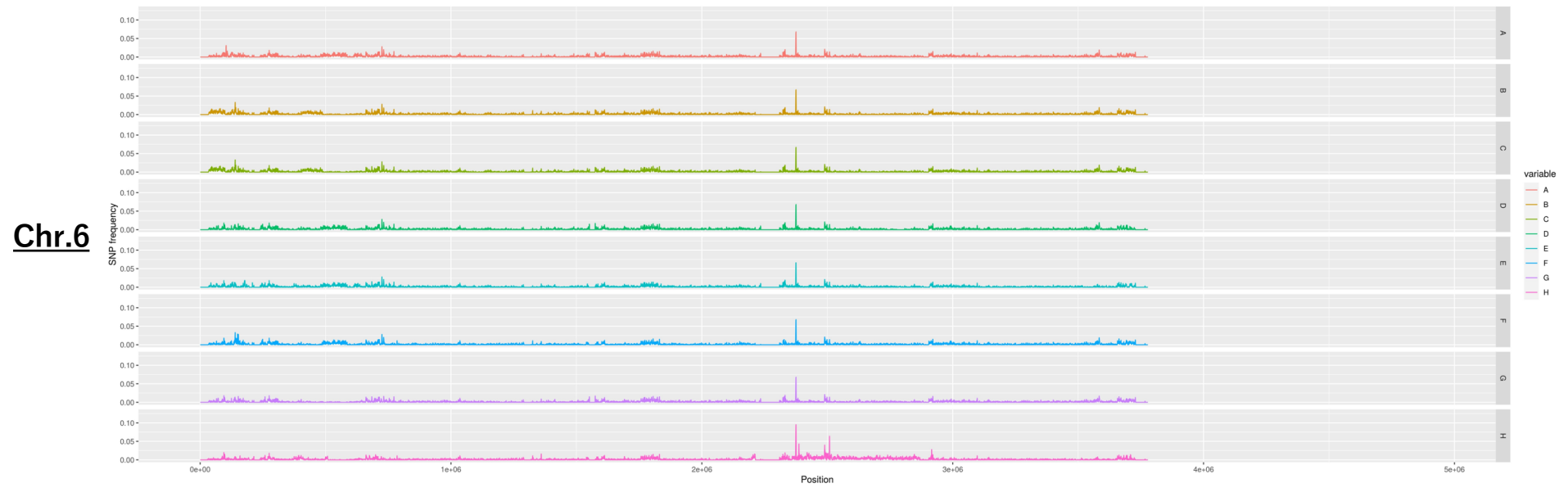

G

Chr.7

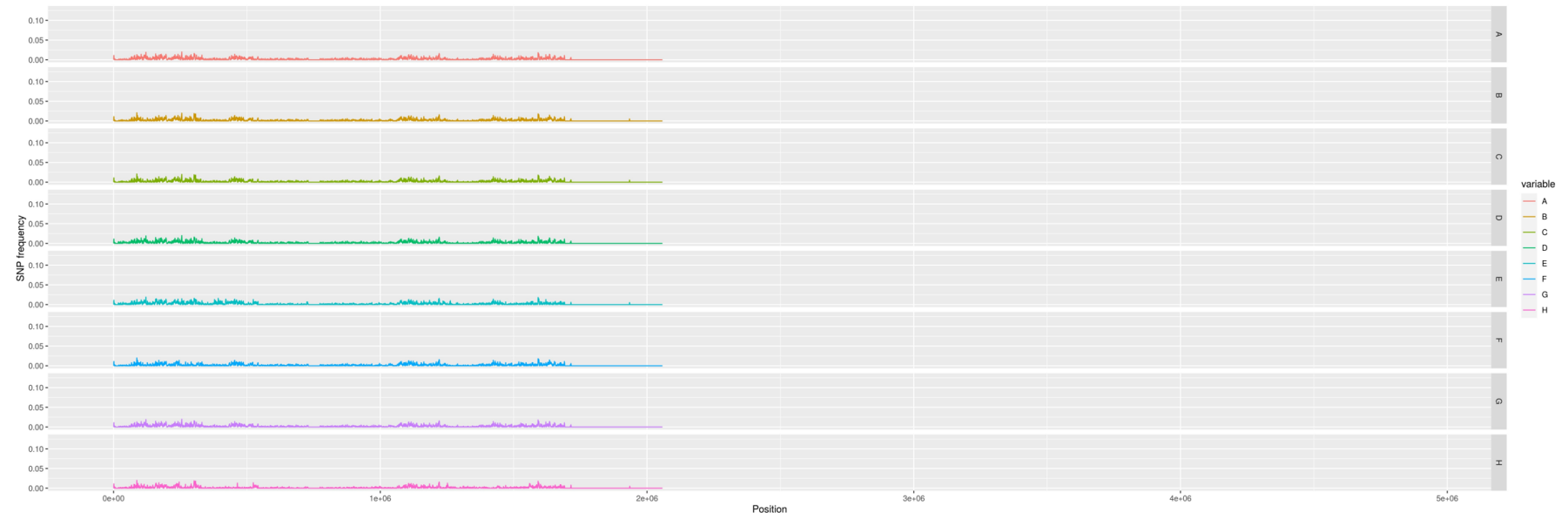

H

Chr.8

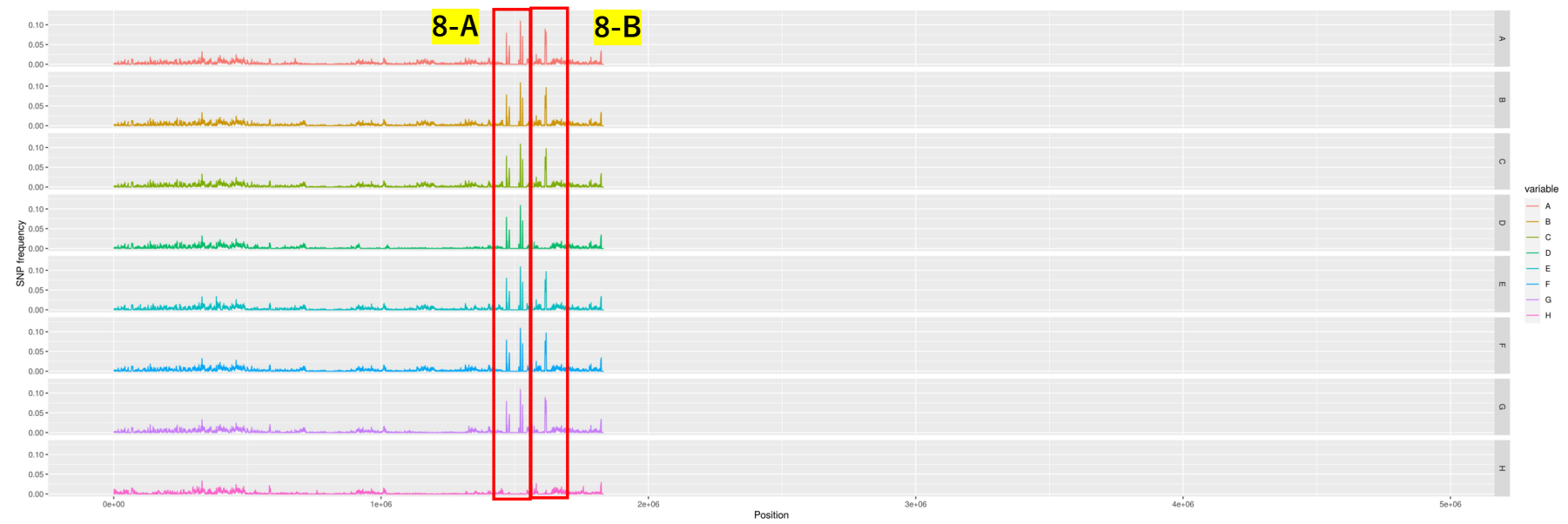

Supplement: Supplementary file 1 — Fig. S1. Genome‐wide SNP frequency compared among the eight strains (3‐1‐A to 3‐1‐H). The 10 regions where the pattern is characteristically distinct among the strains are marked by red boxes. [file EMI-23-5621-s002.pdf]
